# Molecular detection and quantification of Plasmodium falciparum-infected human hepatocytes in chimeric immune-deficient mice

**DOI:** 10.1186/1475-2875-12-430

**Published:** 2013-11-24

**Authors:** Lander Foquet, Cornelus C Hermsen, Geert-Jan van Gemert, Louis Libbrecht, Robert Sauerwein, Philip Meuleman, Geert Leroux-Roels

**Affiliations:** 1 Center for Vaccinology, Ghent University and University Hospital, De Pintelaan 185, Ghent 9000, Belgium; 2 Medical Centre, Radboud University Nijmegen, Geert Grooteplein 28, GA 6525 Nijmegen, The Netherlands; 3 Department of Pathology, Ghent University and University Hospital, De Pintelaan 185, Ghent 9000, Belgium

**Keywords:** Humanized mouse model, Malaria, *Plasmodium falciparum*, Sporozoite, Liver stage, *in vivo*, qPCR

## Abstract

**Background:**

Chimeric mice with humanized livers represent a promising tool for infections with *Plasmodium falciparum* to evaluate novel methods for prevention and treatment of pre-erythrocytic stages. Adequate assessment of hepatic infections is generally compromised by the limited number of human hepatocytes infected by developing parasites.

**Methods:**

A qPCR-based method has been developed that sensitively and reliably detects *P. falciparum* liver stage infection of humanized mice and quantitatively expresses the results as the number of parasites per human hepatocyte.

**Results:**

This assay allows for detection of liver stage parasites after challenging humanized mice with infected mosquito bites or after intravenous injection with sporozoites. The sensitivity of the protocol, which comprises approximately 25% of the total chimeric liver, allows for the detection of a single infected hepatocyte in the analysed tissue.

**Conclusions:**

This method allows for the detection and quantification of *P. falciparum* parasites in chimeric mice repopulated with human hepatocytes. It will be a useful tool when studying the *in vivo* therapeutic and/or prophylactic qualities of novel compounds, small molecules or antibodies directed against the liver stage of *P. falciparum* infections.

## Background

*Plasmodium falciparum* is responsible for most of the estimated 219 million clinical malaria cases that were reported in 2010 [1]. Rodent models for malaria (e.g. *Plasmodium berghei* in mice) are used to test possible new anti-malarial drugs [2] and candidate vaccines [3] before entering the clinical phase of downstream product development [4]. However, significant differences between rodent models and human malaria [5] necessitate the availability of alternative models to study *P. falciparum*. Humanized mouse models have been developed to study either blood stage or liver stage of malaria. By engrafting human red blood cells (huRBC) into immunodeficient mice, a high percentage of huRBC in mouse peripheral blood can be obtained [6]. Subsequent injection of infected human erythrocytes results in a blood stage infection that can be maintained for several days and allows for the *in vivo* testing of new therapies against blood stage infection [7,8]. The liver stage of malaria can be studied in human liver chimeric mice. These models are based on the ability of immunodeficient mice with a severe liver disease to accept a graft of human hepatocytes and allow these cells to home into the diseased mouse liver where their expansion leads to high degrees of repopulation with human hepatocytes. Immune deficient mice have acquired severe liver disease through the transgenic overexpression of urokinase-type plasminogen activator in uPA^+/+^-SCID mice or a knockout of fumarylacetoacetate hydrolase (FAH) in FRG (Fah^-/-^Rag2^-/-^IL2-Rg^-/-^) mice [9-13]. These humanized mice can subsequently be infected with *P. falciparum* sporozoites [14,15] and full parasite maturation in human hepatocytes can be achieved, but emerging parasites cannot successfully infect murine erythrocytes [16]. The Center for Vaccinology (CEVAC) has been successful in producing chimeric uPA^+/+^-SCID mice with a high degree of repopulation with human hepatocytes to study hepatitis B and C virus (HBV and HCV) infections [17-20]. Recently, transfer of human hepatocytes was started in FRG mice that originate from Marcus Grompe’s laboratory [10].

Several methods are available to detect parasites in an infected humanized mouse model. Immune staining techniques do visualize infected hepatocytes but quantification and comparison of animals with different degrees of human hepatocyte repopulation remains difficult. Moreover, these techniques are labour-intensive with low throughput and limited sensitivity [15,21]. Alternatively, a successful infection of the chimeric mouse liver can be directly visualized *in vivo* after challenge with a GFP-luciferase transgenic *P. falciparum* strain [22]. While enabling parasite detection without sacrificing the animal, this technique lacks the sensitivity to visualize early liver stage infection *in vivo*[23]. The lower sensitivity necessitates the infection of humanized mice by intravenous (IV) injection of up to 10^6^ sporozoites, representing an unnaturally high number of parasites [22]. Therefore, a highly sensitive qPCR-based method was improved to detect and quantify hepatic infections of *P. falciparum,* after IV injection of sporozoites or following natural infection via mosquito bites.

## Methods

### Generation of humanized mice

Humanized uPA^+/+^-SCID mice were generated as previously described [9]. Briefly, within two weeks after birth cryopreserved primary human hepatocytes (approximately 10^6^ cells/mouse, purchased from BD Gentest (Erembodegem, Belgium)) were injected in the spleens of uPA^+/+^-SCID mice [24]. Hepatocytes from the same donor were used to allow comparison between the different experiments. Between six weeks after transplantation and up to one week before the infection experiment, human albumin levels in mouse plasma were measured using Human Albumin ELISA Quantitation kit (Bethyl Laboratories Inc, Montgomery, TX, USA). Animals with human albumin levels >2 mg/mL were considered successfully engrafted and used for infection studies. All procedures were approved by the Animal Ethics Committee of the Faculty of Medicine and Health Sciences of the Ghent University.

### Parasite challenge

*Plasmodium falciparum* NF54 infected *Anopheles stephensi* mosquitoes were reared as described previously [25] at Radboud University (Nijmegen, Netherlands). Chimeric mice were infected with sporozoites, either by mosquito bites or by injection in the retro-orbital venous plexus. To mimic a natural infection as closely as possible, humanized mice were exposed for 20 minutes to bites of 20 *P. falciparum-*infected mosquitoes that contained each on average 70,000 sporozoites in the salivary gland. Before exposure to the mosquitoes, abdomen and chest of the mice were shaven with electric clippers. Next mice were positioned on a cardboard box containing 20 infected mosquitoes (one box for each mouse). Successful blood feeding (mean: 17 mosquitoes) and sporozoite presence (100%) was confirmed by mosquito dissection after the challenge experiment [3,26]. To inject a defined number of parasites, sporozoites were extracted by hand dissection from the salivary glands of infected mosquitoes killed by ethanol spraying [23]. Mice were anesthetised with 3% isoflurane in 0.5 L/min 100% O_2_ and 150,000 salivary gland sporozoites in 100 μl RPMI 1640 (GIBCO) were injected in the retro-orbital venous plexus [27], because the extremely small diameter of the tail vein of humanized uPA^+/+^-SCID mice precludes intravenous injection.

### Isolation of DNA and quantitation of plasmodium falciparum and human hepatocyte DNA

**Figure 1:**
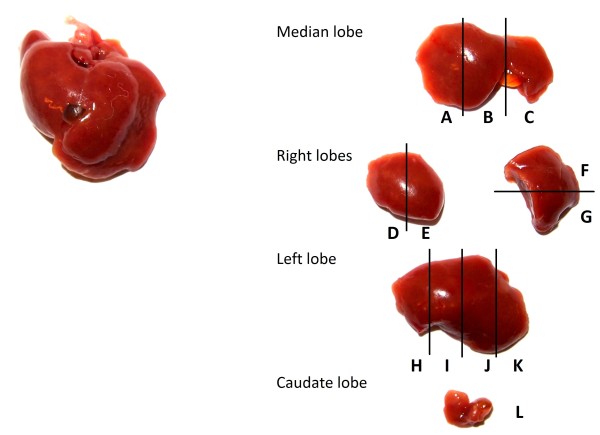
**Standardizing the sampling of humanized liver fragments.** Five days post-infection, mice are euthanized by cervical dislocation. The livers are carefully removed, rinsed in PBS and 12 standardized fragments **(A-L)** are prepared and placed in RNALater until further analysis.

**Figure 2:**
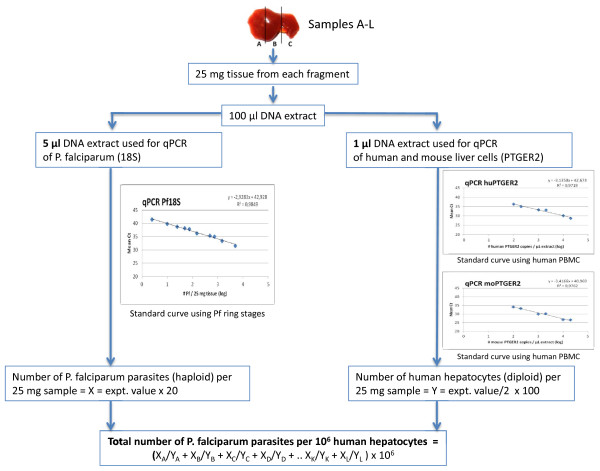
**Overview of assay procedures.** Preparation of liver tissue sections, tissue fragmentation, DNA isolation, qPCR and calculations to obtain a normalized determination of *P. falciparum* parasite numbers (left panel) in a humanized liver (right panels).

**Figure 3:**
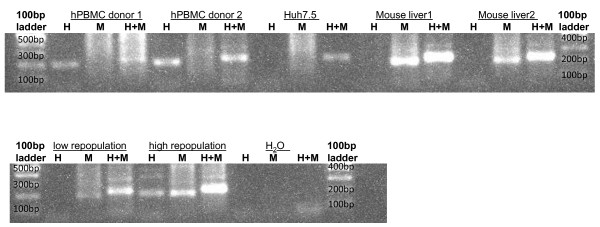
**Species specificity of primers used for the detection of human and mouse DNA.** H: human-specific forward primer, contains the FAM-probe binding site; M: mouse-specific forward primer, contains the Cy5-binding site; H + M: human and mouse common forward primer, used in qPCR experiments. A human and mouse common reverse primer was used in all reactions. Human PBMC’s and SCID mouse liver extract give rise to the human and mouse specific PCR products. The human cell line Huh7.5 does not lead to a human or mouse specific PCR product. Not transplanted mice or low repopulated chimeric mice do not induce a human specific PCR product, whereas a good repopulated chimeric mouse has both human and mouse specific bands. This confirms that the primers used are specific for the mouse and/or human PTGER2 gene expressed in a chimeric mouse liver.

Five days after infection mice were maximally bled (around 500 μL) and subsequently euthanized by cervical dislocation. The livers were carefully removed, cut into 12 standardized sections and stored in RNALater (Ambion) at 4°C until analysis (Figure 1). Of each section, exactly 25 mg (± 0.1 mg) tissue was taken for DNA extraction in 100 μL elution buffer with the High Pure PCR Template Preparation Kit (Roche). *Plasmodium falciparum* DNA levels were quantified using a highly sensitive qPCR assay [28]. Briefly, 5 μL DNA extract was added to 20 μL mastermix (LightCycler 480 Probes Master, Roche) containing *P. falciparum* 18SRNA gene-specific primers and a probe labelled with 6-carboxy-fluorescein (FAM) as a reporter and 6-carboxy-tetramethylrhodamine (TAMRA) as a quencher. *Plasmodium falciparum* standard curves were prepared by spiking DNA extracts from titrated samples of ring stage *P. falciparum-*infected erythrocytes (range 10–10,000 parasites (Pf) per 100 μL extract) with DNA extracts from a non-infected humanized liver. The detection limit was set at 0.2 Pf/5 μL extract, since the sensitivity of this standardized assay (20Pf/mL blood) correlates to 0.2 Pf/5 μL extract as shown in Figure 2. Because the reaction is performed in duplicate it is possible to detect 0.2 Pf in 10% of the total extract (if only one out of two reactions is positive). Since all 12 fragments are analysed separately but in an identical way it is possible to detect two Pf in 300 mg tissue (this if only one of twelve fragments is positive). To assess the repopulation of chimeric livers with human hepatocytes and to express the *P. falciparum* infection as a number of parasites per 10^6^ human hepatocytes, employed a qPCR was employed as described by Alcoser *et al.,* that quantifies the relative amount of human and mouse cells in mixed tissues [29]. Briefly, 1 μL DNA extract was added to 19 μL mastermix (LightCycler 480 Probes Master, Roche) containing common primers that amplify a 215 bp region located in the human and mouse prostaglandin E receptor 2 (PTGER2) genes (Figure 3). The amplified region contains a non-homologous sequence which is targeted by two species-specific Taqman probes, each conjugated with a unique fluorescent tag (FAM and Cy5), which makes it possible to quantify the copy number and differentiate between mouse and human origin in duplex. Standard curves were prepared by DNA extraction from a titration of defined numbers of human PBMC and mouse splenocytes (Figure 2). Percentage calculation was verified by making various ratios of mouse and human DNA extracts.

### Liver tissue specimens and histochemistry

Liver samples were fixed in formalin and slides were stained with haematoxylin-eosin following standard techniques as described [9].

## Results

### Standardizing the sampling of humanized liver fragments

**Figure 4:**
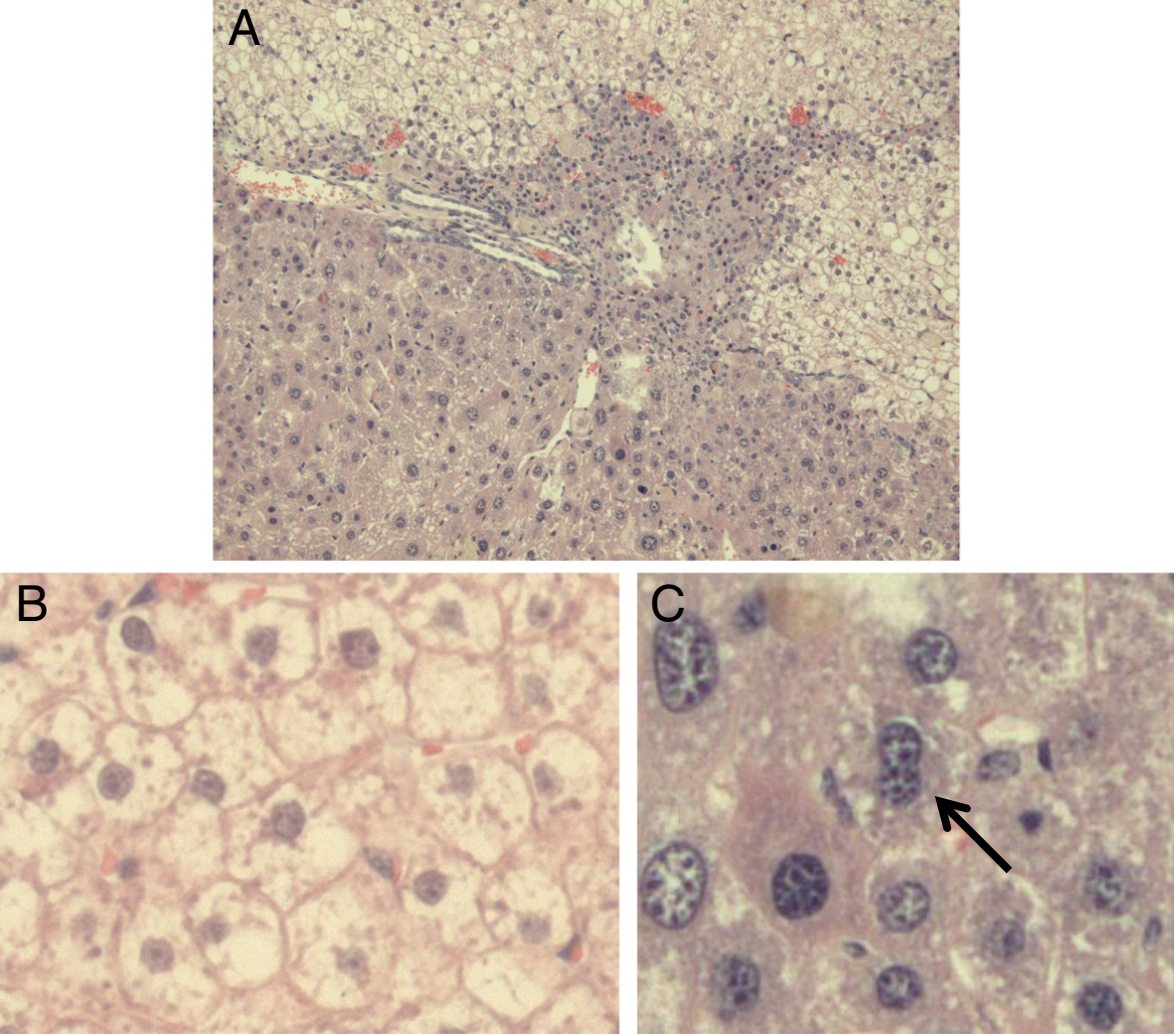
**Histology and determination of polyploidy of a chimeric liver. (A)** A representative section of a chimeric liver (haematoxylin and eosin staining) showing the morphological differences between pale human hepatocytes (upper zone), diseased mouse parenchyma (middle zone) and healthy mouse parenchyma originating from mouse hepatocytes that have eliminated both uPA transgene copies, known as red nodules (lower zone). Detailed view of chimeric liver sections, showing human hepatocytes **(B)** none of which is multinucleated and mouse hepatocytes **(C)**, one of which is binucleated (arrow). Original magnification A: 200 x, B and C: 400 x.

Injection of *P. falciparum* sporozoites in a chimeric mouse will result in infection of an unknown and low number of infected human hepatocytes distributed in a large background of non-infected mouse and human cells (Figure 4). Consequently, an analysis of the complete liver by cell lysis and subsequent DNA extraction will result in a strong dilution of parasitic DNA and loss of sensitivity. Therefore, exactly 25 mg of liver tissue was sampled from 12 standardized sections (Figure 1) for DNA extraction in a volume of 100 μL. Five μL of each extract was used in duplicate to measure the number of parasites by qPCR and 1 μL to determine the fraction (percentage) of human hepatocytes in each fragment (Figure 2). This approach allowed for the analysis of several millions of human hepatocytes randomly distributed in each chimeric liver (Additional file 1: Table S1).

### Measurement of parasites in humanized livers: standardization and quantification

Both presence of parasite [28] as well as mouse and human [29] DNA was determined in each fragment by qPCR (Figures 2 and 3). Since hepatocytes are frequently multinucleated [30], a histological examination was performed of humanized liver sections of two chimeric mice as described before [12,31]. Because less than 10% of both human and mouse hepatocytes displayed a multinucleated aspect (Figure 4), the calculation of the number of hepatocytes was not corrected. The ratio of human DNA *versus* [human DNA + mouse DNA] was multiplied by 1.7 because it is described [13,32] that only 60% of chimeric liver tissue consists of hepatocytes (40% are other cell types) (Additional file 1: Table S1). Calculating the percentage of human hepatocytes in the chimeric liver by using the qPCR data made it possible to compare the degree of repopulation by human hepatocytes of each liver fragment and each liver as a whole. The degree of repopulation measured in the separate fragments corresponds well with the average degree (Additional file 1: Table S1). Finally, the liver parasite burden, expressed as the number of parasites per million human hepatocytes was calculated by dividing the total number of parasites detected in the 12 samples derived from a chimeric liver by the total number of human hepatocytes in these samples (Figure 2 and Additional file 1: Table S1).

### Comparison of different infection protocols

After the injection of 150,000 *P. falciparum* sporozoites in the retro-orbital venous plexus [14,15], infection of human hepatocytes was demonstrated in all mice (mean: 11,405.6 parasites per 10^6^ human hepatocytes) using the analytical methods described above (Additional file 2: Table S2). In addition, humanized mice were exposed to 20 infected mosquitoes during 20 minutes [33]. Additional file 1: Table S1 shows that the ensuing infection can be detected in multiple samples (A-L) and is more comparable in the different animals irrespective of their degree of chimerism than when mice were challenged by injection of 150,000 *P. falciparum* sporozoites (mean: 1,503.1 parasites per 10^6^ human hepatocytes). The observed lower variation in the liver parasite burden was not anticipated since mosquito bites lead to an unknown and variable parasite challenge [34].

## Discussion

A method is presented to detect and quantify *P. falciparum* parasites in the livers of humanized mice following infection via intravenous injection of sporozoites or bites from infected mosquitoes. The approach combines a systematic analysis of 12 standardized liver sections from each chimeric liver to avoid sampling bias and sample dilution with the analytical sensitivities of qPCR methods to quantify parasite numbers and human hepatocyte content of each fragment.

Based on qPCR results obtained by sacrificing humanized mice at different time points, it is estimated that a single infected hepatocyte may contain approximately 20 parasites three days after infection and between 200 and 400 parasites five days after infection. Previous studies estimate that this number could rise even further to more than 30,000 parasites per infected hepatocyte at the end of the pre-erythrocytic stage, six to seven days after infection [15,35]. Humanized mice were sacrificed five days after infection because this leaves sufficient time for parasite DNA not confined to an infected human hepatocyte to be cleared, while this point in time is sufficiently remote from the beginning of the end of the liver stage at day 6 to prevent the release of merozoites from infected hepatocytes and subsequent clearance from the blood since these cannot infect mouse erythrocytes [15,28].

Analysis of multiple (12) samples from each liver increases the sensitivity of the method as compared to the analysis of a small sample taken from a homogenized whole liver. As few as two parasites (Pf) in 300 mg analysed tissue can be detected. Indeed, the latter approach may lead to a dilution that is possibly too high to detect DNA of parasites originating from only a few infected cells located within a limited zone of the liver. If one assumes that only one human hepatocyte was infected with *P. falciparum*, this would result in at least 200 Pf after five days. Since approximately one quarter of the total liver was analysed (the liver represents 9% of the total body weight), there was 25% chance that the infected hepatocyte is located within the tissue analysed. Since that hepatocyte would be present in one of the 12 fragments, this one fragment would contain at least 200 Pf. Given that the assay has a sensitivity of two parasites per fragment, the parasites enclosed in a single infected hepatocyte will be detected. This is considerably better than any other method currently available for the *in vivo* study of *Plasmodium falciparum* infection (e g, histology, *in vivo* bioluminescence with luciferase expressing parasites, etc.).

**Figure 5:**
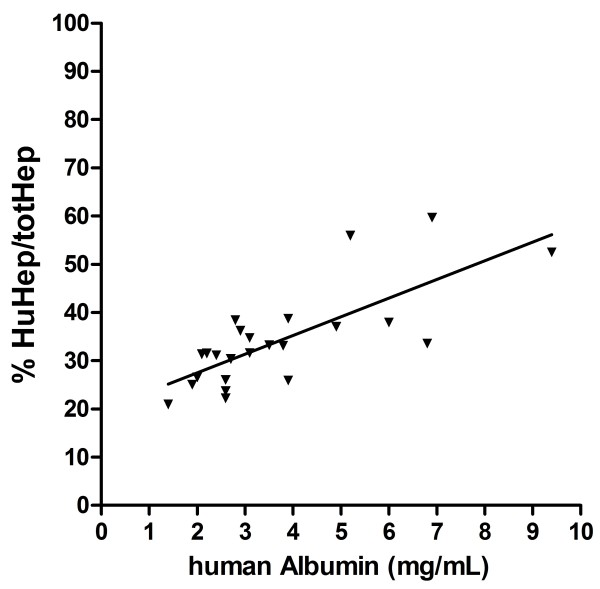
**correlation human albumin and percentage repopulation.** The calculated percentage repopulation (%huHEP/totHEP) is plotted against the measured human albumin plasma concentrations for the animals described in Additional file 2: Table S2.

Quantification of both human and mouse DNA in addition to *P. falciparum* DNA allowed for the determination of the degree (percentage) of human repopulation of each of the 12 mouse liver fragments (Additional file 1: Table S1). This approach differs from previous studies in which the degree of chimerism was calculated by determining the proportion of the liver parenchyma surface area that consisted of human hepatocytes based on immunohistochemical analyses of a series of liver sections [11,31,36]. Since hepatocytes are much larger than non-parenchymal cells, the area occupied by human hepatocytes will overestimate the fraction represented as cell numbers. The data show that the percentage of repopulation of a humanized mouse in different liver lobes is quite stable and does not significantly influence the infection with *P. falciparum* sporozoites (Additional file 2: Table S2). Figure 5 shows that the percentage human hepatocytes correlates well with the human albumin concentrations in chimeric mice (R = 0.57; P < 0.0001).

Normalizing the liver parasite burden of each animal, and expressing this as number of parasites per million human hepatocytes, enables the conduct of comparative studies while using animals with different degrees of chimerism. This is important since the generation of humanized mice is a complex, expensive and time consuming process and the resulting chimeric livers have different degrees of human hepatocyte repopulation.

The data here shows that infected mosquito bites lead to a detectable infection in chimeric mice that is at least as reproducible as IV injection of 150,000 sporozoites isolated from salivary glands of infected mosquitoes (Additional file 2: Table S2). Using the estimation that one mosquito blood meal results in the injection of up to 500 sporozoites [34], exposure to 20 infected mosquitoes can possibly result in the deposition of ± 10,000 sporozoites into the skin. This is still 15-fold less than an IV injection of 150,000 sporozoites and 100-fold less than the inoculum (10^6^ sporozoites) previously injected by other investigators [14,15]. The results show that an IV injection of 150,000 sporozoites leads only to an average 7.5-fold increase in detected parasites compared to exposure to 20 infected mosquitoes (Additional file 2: Table S2). The cause of this discrepancy is not known, but could be attributed to sporozoites killed during the dissection process.

Therefore the natural infection route for challenge may be preferable to study early events during *P. falciparum* infections for a number of reasons: i) material injected intravenously also contains an undefined amount of debris from the dissection of the salivary glands from the mosquitoes and may compromise success of infection and the ensuing processes; ii) IV injection of sporozoites bypasses a series of natural events (epithelial crossing, skin cell reactivity, etc.) of which the precise biological impact and necessity for hepatocyte recognition and invasion are not yet defined. In this way, possible effects of tested novel compounds or anti-sporozoite antibodies will be missed; and, iii) for the evaluation of agents that may have an effect on sporozoites migration to the liver, the duration of contact prior to liver invasion can be of importance. This contact time is much longer when sporozoites are deposited in the host’s skin by a mosquito bite (minutes) compared to direct deposition in the bloodstream [34,37].

## Conclusions

A protocol was developed to detect and quantify *P. falciparum* parasites to study the therapeutic and/or prophylactic qualities of novel compounds, small molecules or antibodies, in chimeric mice repopulated with human hepatocytes. Apart from being susceptible to human hepatotropic microorganisms these mice also express a humanized drug metabolism [38-42]. This will accelerate and reduce the costs of the development of new strategies to prevent and treat the pre-erythrocytic stage of malaria before these are tested in human volunteers.

## Abbreviations

ELISA: Enzyme-linked immunosorbent assay; Fah: Fumarylacetoacetate hydrolase; FAM: 6-carboxy-fluorescein; FRG: Fah^-/-^Rag2^-/-^IL2-Rg^-/-^; GFP: Green fluorescent protein; HBV: Hepatitis B virus; HCV: Hepatitis C virus; huRBC: Human red blood cells; IL2-Rg: Interleukin-2 receptor g chain; IV: Intravenous; NF54: Nijmegen falciparum strain 54; PBMC: Peripheral blood mononuclear cell; PTGER2: Prostaglandin E receptor 2; qPCR: Quantitative polymerase chain reaction; RAG: Recombination activating gene; SCID: Severe combined immunodeficiency; TAMRA: 6-carboxy-tetramethylrhodamine; uPA: Urokinase plasminogen activator.

## Competing interests

The authors have declared that they have no competing interests.

## Authors’ contributions

Conception and design of the experiments: LF, CCH, GJVG, RS, PM, GLR; data acquisition: LF; data analysis: LF, CCH, RS, PM, GLR. All authors contributed to the development of the manuscript, were involved in finalizing the manuscript, all authors read and approved the final manuscript.

## Supplementary Material

Additional file 1: Table S1 Analysis of the livers of humanized mice after IV sporozoites injection. Analysis of two infected humanized mice. Panel 1: number of detected parasites in twelve samples (five μL from each) and mean. Panel 2: percentage of human hepatocytes in twelve samples, mean, and percentage repopulation after correction for the percentage of the liver that is made up of hepatocytes. Panel 3: total number of human hepatocytes analysed in each sample, total number human hepatocytes analysed for each mouse, and the liver parasite burden (expressed as the number of parasites per million human hepatocytes) calculated from panel 1 and 3. Click here for file

Additional file 2: Table S2 List of sporozoite injections and mosquito challenges in humanized mice. Twelve mice were infected by intravenous sporozoite injection or by mosquito challenge. Panel 1: human albumin measured by ELISA, percentage repopulation calculated by qPCR, total number of human hepatocytes analysed per mouse, number of parasites in twelve samples (five μL from each), mean, and liver parasite burden (expressed as the number of parasites per million human hepatocytes). Panel 2: for each infection route or both combined: mean weight, mean albumin, mean percentage repopulation, mean number of human hepatocytes analysed, percentage of positive samples for each sampling site in the humanized livers, mean number of parasites in each sample (five μL from each), mean liver parasite burden (expressed as the number of parasites per million human hepatocytes). Click here for file

## References

[B1] WHO World malaria report 2012 2012 Geneva: World Health Organization

[B2] Booker ML Bastos CM Kramer ML Barker RH Jr Skerlj R Sidhu AB Deng X Celatka C Cortese JF Guerrero Bravo JE Crespo Llado KN Serrano AE Angulo-Barturen I Jimenez-Diaz MB Viera S Garuti H Wittlin S Papastogiannidis P Lin JW Janse CJ Khan SM Duraisingh M Coleman B Goldsmith EJ Phillips MA Munoz B Wirth DF Klinger JD Wiegand R Sybertz E Novel inhibitors of *Plasmodium falciparum* dihydroorotate dehydrogenase with anti-malarial activity in the mouse model J Biol Chem 2010 285 33054 33064 20702404 10.1074/jbc.M110.162081 PMC2963363

[B3] Leitner WW Bergmann-Leitner ES Angov E Comparison of Plasmodium berghei challenge models for the evaluation of pre-erythrocytic malaria vaccines and their effect on perceived vaccine efficacy Malar J 2010 9 145 20507620 10.1186/1475-2875-9-145 PMC2904356

[B4] Duffy PE Sahu T Akue A Milman N Anderson C Pre-erythrocytic malaria vaccines: identifying the targets Expert Rev Vaccines 2012 11 1261 1280 23176657 10.1586/erv.12.92 PMC3584156

[B5] Macchiarini F Manz MG Palucka AK Shultz LD Humanized mice: are we there yet? J Exp Med 2005 202 1307 1311 16301740 10.1084/jem.20051547 PMC2212979

[B6] Angulo-Barturen I Jimenez-Diaz MB Mulet T Rullas J Herreros E Ferrer S Jimenez E Mendoza A Regadera J Rosenthal PJ Bathurst I Pompliano DL Gomez De Las Heras F Gargallo-Viola D A murine model of falciparum-malaria by in vivo selection of competent strains in non-myelodepleted mice engrafted with human erythrocytes PLoS One 2008 3 e2252 18493601 10.1371/journal.pone.0002252 PMC2375113

[B7] Jimenez-Diaz MB Mulet T Viera S Gomez V Garuti H Ibanez J Alvarez-Doval A Shultz LD Martinez A Gargallo-Viola D Angulo-Barturen I Improved murine model of malaria using Plasmodium falciparum competent strains and non-myelodepleted NOD-scid IL2Rgammanull mice engrafted with human erythrocytes Antimicrob Agents Chemother 2009 53 4533 4536 19596869 10.1128/AAC.00519-09 PMC2764183

[B8] Arnold L Tyagi RK Meija P Swetman C Gleeson J Pérignon J-L Druilhe P Further improvements of the P. Falciparum humanized mouse model PLoS One 2011 6 e18045 21483851 10.1371/journal.pone.0018045 PMC3069031

[B9] Meuleman P Libbrecht L De Vos R de Hemptinne B Gevaert K Vandekerckhove J Roskams T Leroux-Roels G Morphological and biochemical characterization of a human liver in a uPA-SCID mouse chimera Hepatology 2005 41 847 856 15791625 10.1002/hep.20657

[B10] Azuma H Paulk N Ranade A Dorrell C Al-Dhalimy M Ellis E Strom S Kay MA Finegold M Grompe M Robust expansion of human hepatocytes in Fah^-/-^/Rag2^-/-^/Il2rg^-/-^ mice Nat Biotechnol 2007 25 903 910 17664939 10.1038/nbt1326 PMC3404624

[B11] Bissig KD Le TT Woods NB Verma IM Repopulation of adult and neonatal mice with human hepatocytes: a chimeric animal model Proc Natl Acad Sci USA 2007 104 20507 20511 18077355 10.1073/pnas.0710528105 PMC2154461

[B12] Vanwolleghem T Libbrecht L Hansen BE Desombere I Roskams T Meuleman P Leroux-Roels G Factors determining successful engraftment of hepatocytes and susceptibility to hepatitis B and C virus infection in uPA-SCID mice J Hepatol 2010 53 468 476 20591528 10.1016/j.jhep.2010.03.024

[B13] Tateno C Miya F Wake K Kataoka M Ishida Y Yamasaki C Yanagi A Kakuni M Wisse E Verheyen F Inoue K Sato K Kudo A Arii S Itamoto T Asahara T Tsunoda T Yoshizato K Morphological and microarray analyses of human hepatocytes from xenogeneic host livers Lab Invest 2013 93 54 71 23147226 10.1038/labinvest.2012.158

[B14] Sacci JB Jr Alam U Douglas D Lewis J Tyrrell DL Azad AF Kneteman NM Plasmodium falciparum infection and exoerythrocytic development in mice with chimeric human livers Int J Parasitol 2006 36 353 360 16442544 10.1016/j.ijpara.2005.10.014

[B15] Vaughan AM Mikolajczak SA Wilson EM Grompe M Kaushansky A Camargo N Bial J Ploss A Kappe SH Complete Plasmodium falciparum liver-stage development in liver-chimeric mice J Clin Invest 2012 122 3618 3628 22996664 10.1172/JCI62684 PMC3461911

[B16] Vaughan AM Kappe SH Ploss A Mikolajczak SA Development of humanized mouse models to study human malaria parasite infection Future Microbiol 2012 7 657 665 22568719 10.2217/fmb.12.27 PMC3848604

[B17] Meuleman P Leroux-Roels G The human liver-uPA-SCID mouse: a model for the evaluation of antiviral compounds against HBV and HCV Antiviral Res 2008 80 231 238 18706933 10.1016/j.antiviral.2008.07.006

[B18] Bukh J Meuleman P Tellier R Engle RE Feinstone SM Eder G Satterfield WC Govindarajan S Krawczynski K Miller RH Leroux-Roels G Purcell RH Challenge pools of hepatitis C virus genotypes 1–6 prototype strains: replication fitness and pathogenicity in chimpanzees and human liver-chimeric mouse models J Infect Dis 2010 201 1381 1389 20353362 10.1086/651579 PMC2941994

[B19] Meuleman P Bukh J Verhoye L Farhoudi A Vanwolleghem T Wang RY Desombere I Alter H Purcell RH Leroux-Roels G In vivo evaluation of the cross-genotype neutralizing activity of polyclonal antibodies against hepatitis C virus Hepatology 2011 53 755 762 21319203 10.1002/hep.24171 PMC3079546

[B20] Meuleman P Teresa Catanese M Verhoye L Desombere I Farhoudi A Jones CT Sheahan T Grzyb K Cortese R Rice CM Leroux-Roels G Nicosia A A Human monoclonal antibody targeting scavenger receptor class B type I precludes hepatitis C virus infection and viral spread in vitro and in vivo Hepatology 2012 55 364 372 21953761 10.1002/hep.24692 PMC3262867

[B21] Sacci J Schriefer M Resau J Wirtz R Detolla L Markham R Azad A Mouse model for exoerythrocytic stages of Plasmodium falciparum malaria parasite Proc Natl Acad Sci USA 1992 89 3701 3706 1570289 10.1073/pnas.89.9.3701 PMC525558

[B22] Vaughan A Mikolajczak S Camargo N Lakshmanan V Kennedy M Lindner S Miller J Hume JC Kappe S A transgenic Plasmodium falciparum NF54 strain that expresses GFP-luciferase throughout the parasite life cycle Mol Biochem Parasitol 2012 186 143 147 23107927 10.1016/j.molbiopara.2012.10.004

[B23] Ploemen IH Prudencio M Douradinha BG Ramesar J Fonager J van Gemert GJ Luty AJ Hermsen CC Sauerwein RW Baptista FG Mota MM Waters AP Que I Lowik CW Khan SM Janse CJ Franke-Fayard BM Visualisation and quantitative analysis of the rodent malaria liver stage by real time imaging PLoS One 2009 4 e7881 19924309 10.1371/journal.pone.0007881 PMC2775639

[B24] Meuleman P Vanlandschoot P Leroux-Roels G A simple and rapid method to determine the zygosity of uPA-transgenic SCID mice Biochem Biophys Res Commun 2003 308 375 378 12901879 10.1016/s0006-291x(03)01388-3

[B25] Ponnudurai T Lensen AH Van Gemert GJ Bensink MP Bolmer M Meuwissen JH Infectivity of cultured Plasmodium falciparum gametocytes to mosquitoes Parasitology 1989 98 Pt 2 165 173 2668861 10.1017/s0031182000062065

[B26] Yoeli M Upmanis RS Vanderberg J Most H Life cycle and patterns of development of Plasmodium berghei in normal and experimental hosts Mil Med 1966 131 Suppl 900 914 4380325

[B27] Yardeni T Eckhaus M Morris H Huizing M Hoogstraten-Miller S Retro-orbital injections in mice Lab Anim (NY) 2011 40 155 21508954 10.1038/laban0511-155 PMC3158461

[B28] Hermsen CC Telgt DS Linders EH van de Locht LA Eling WM Mensink EJ Sauerwein RW Detection of Plasmodium falciparum malaria parasites in vivo by real-time quantitative PCR Mol Biochem Parasitol 2001 118 247 251 11738714 10.1016/s0166-6851(01)00379-6

[B29] Alcoser SY Kimmel DJ Borgel SD Carter JP Dougherty KM Hollingshead MG Real-time PCR-based assay to quantify the relative amount of human and mouse tissue present in tumor xenografts BMC Biotechnol 2011 11 124 22176647 10.1186/1472-6750-11-124 PMC3281124

[B30] Dufour JF Clavien PA Signaling pathways in liver diseases 2010 Heidelberg: Springer

[B31] Meuleman P Libbrecht L de Vos R Roskams T Leroux-Roels G Cytopathic effects of hepatitis B virus infection in long-term infected chimeric uPA-SCID mice Hepatology 2004 40 267a

[B32] Marcos R Monteiro RA Rocha E Design-based stereological estimation of hepatocyte number, by combining the smooth optical fractionator and immunocytochemistry with anti-carcinoembryonic antigen polyclonal antibodies Liver Int 2006 26 116 124 16420517 10.1111/j.1478-3231.2005.01201.x

[B33] Sauerwein RW Roestenberg M Moorthy VS Experimental human challenge infections can accelerate clinical malaria vaccine development Nat Rev Immunol 2011 11 57 64 21179119 10.1038/nri2902

[B34] Jin Y Kebaier C Vanderberg J Direct microscopic quantification of dynamics of plasmodium berghei sporozoite transmission from mosquitoes to mice Infect Immun 2007 75 5532 5539 17785479 10.1128/IAI.00600-07 PMC2168273

[B35] Garnham PCC Malaria parasites and other haemosporidia 1966 Oxford: Blackwell Scientific

[B36] Tateno C Yoshizane Y Saito N Kataoka M Utoh R Yamasaki C Tachibana A Soeno Y Asahina K Hino H Asahara T Yokoi T Furukawa T Yoshizato K Near completely humanized liver in mice shows human-type metabolic responses to drugs Am J Pathol 2004 165 901 913 15331414 10.1016/S0002-9440(10)63352-4 PMC1618591

[B37] Vanderberg J Frevert U Intravital microscopy demonstrating antibody-mediated immobilisation of Plasmodium berghei sporozoites injected into skin by mosquitoes Int J Parasitol 2004 34 991 996 15313126 10.1016/j.ijpara.2004.05.005

[B38] Lootens L Meuleman P Pozo OJ Van Eenoo P Leroux-Roels G Delbeke FT uPA^+/+^-SCID mouse with humanized liver as a model for in vivo metabolism of exogenous steroids: methandienone as a case study Clin Chem 2009 55 1783 1793 19643840 10.1373/clinchem.2008.119396

[B39] Pozo OJ Van Eenoo P Deventer K Lootens L Van Thuyne W Parr MK Schanzer W Sancho JV Hernandez F Meuleman P Leroux-Roels G Delbeke FT Detection and characterization of a new metabolite of 17alpha-methyltestosterone Drug Metab Dispos 2009 37 2153 2162 19704028 10.1124/dmd.109.028373

[B40] Yoshizato K Tateno C In vivo modeling of human liver for pharmacological study using humanized mouse Expert Opin Drug Metab Toxicol 2009 5 1435 1446 19715443 10.1517/17425250903216664

[B41] Strom SC Davila J Grompe M Chimeric mice with humanized liver: tools for the study of drug metabolism, excretion, and toxicity Meth Mol Biol 2010 640 491 509 10.1007/978-1-60761-688-7_27 PMC3136240 20645070

[B42] De Serres M Bowers G Boyle G Beaumont C Castellino S Sigafoos J Dave M Roberts A Shah V Olson K Patel D Wagner D Yeager R Serabjit-Singh C Evaluation of a chimeric (uPA^+/+^)/SCID mouse model with a humanized liver for prediction of human metabolism Xenobiotica 2011 41 464 475 21370990 10.3109/00498254.2011.560295

